# Iron metabolism and ferroptosis in type 2 diabetes mellitus and complications: mechanisms and therapeutic opportunities

**DOI:** 10.1038/s41419-023-05708-0

**Published:** 2023-03-08

**Authors:** Runyu Miao, Xinyi Fang, Yanjiao Zhang, Jiahua Wei, Yuxin Zhang, Jiaxing Tian

**Affiliations:** 1 Institute of Metabolic Diseases, Guang’ anmen Hospital, China Academy of Chinese Medical Sciences, Beijing, 100053 China; 2 Graduate College, Beijing University of Chinese Medicine, Beijing, China; 3 Graduate College, Changchun University of Chinese Medicine, Changchun, China

**Keywords:** Cell death, Pharmacology

## Abstract

The maintenance of iron homeostasis is essential for proper endocrine function. A growing body of evidence suggests that iron imbalance is a key factor in the development of several endocrine diseases. Nowadays, ferroptosis, an iron-dependent form of regulated cell death, has become increasingly recognized as an important process to mediate the pathogenesis and progression of type 2 diabetes mellitus (T2DM). It has been shown that ferroptosis in pancreas β cells leads to decreased insulin secretion; and ferroptosis in the liver, fat, and muscle induces insulin resistance. Understanding the mechanisms concerning the regulation of iron metabolism and ferroptosis in T2DM may lead to improved disease management. In this review, we summarized the connection between the metabolic pathways and molecular mechanisms of iron metabolism and ferroptosis in T2DM. Additionally, we discuss the potential targets and pathways concerning ferroptosis in treating T2DM and analysis the current limitations and future directions concerning these novel T2DM treatment targets.

## Introduction

Iron is an important trace element for living organisms [1] as it participates in a range of metabolic processes, such as oxygen transport, energy metabolism, nucleotide synthesis, and electron transport [2]. Although vital, excessive amounts of iron can be toxic, therefore, its concentration needs to be maintained within an ideal range. Iron homeostasis is regulated and maintained by iron metabolism. Thus, iron metabolic homeostasis is required for the optimal functioning of fundamental physiological processes [3]. Iron homeostasis in humans is regulated by balancing iron uptake with intracellular utilization and storage. Dietary iron is absorbed by duodenal enterocytes (section 3.1) and binds to transferrin in the plasma. Transferrin limits the production of toxic free radicals and is responsible for ferric-ion delivery into cells. The iron homeostasis system maintains transferrin saturation at physiological levels. During iron metabolism, less than 10% of the iron demand is met by intestinal absorption, and the remaining iron is exported by ferroportin [4]. Ferroportin is regulated by hepcidin, which is a peptide hormone and is often secreted by hepatocytes [5]. Abnormal iron metabolism mainly presents as iron deficiency or overload [3], which triggers multiple pathological changes such as ferroptosis. Ferroptosis is an iron-dependent form of non-apoptotic cell death [6], characterized by iron overload [7] and lipid hydroperoxides accumulation [8].

Numerous studies have shown that ferroptosis plays an important role in the development and progression of type 2 diabetes mellitus (T2DM) and complications. T2DM is a serious global health concern. Physical inactivity and an unhealthy diet are the major T2DM risk factors, and an increasing disease prevalence is observed in children and younger adults [9]. High levels of ferroptosis, mediated by multiple metabolic pathways and signals, can lead to insulin resistance (IR), abnormal metabolism in the liver and fat, and neurological and vascular diseases. Human blood glucose homeostasis is primarily regulated by insulin and glucagon, which promote glycogen synthesis and breakdown, respectively. It has been shown that iron metabolism is involved in different processes of human glucose metabolism such as insulin secretion [10], liver metabolism [11], and fat metabolism [12], and maintains blood glucose homeostasis in multiple organs and tissues.

Recently, scholars gradually found a relationship between iron metabolism and glucose homeostasis [13]. However, studies were largely limited to animal models, and only a few clinical trials were conducted. In this review, we discuss the iron metabolism processes that are involved in glucose homeostasis, explore potential drug targets related to ferroptosis in T2DM and its complications, and list drugs or small molecules that may inhibit ferroptosis by targeting T2DM and its complications. This review provides a basis for a potential treatment approach and its potential clinical applications.

## Molecular mechanism and metabolic basis of ferroptosis

Ferroptosis is a newly recognized type of iron-dependent cell death, characterized by iron overload and lipid peroxidation accumulation. In 2003, Dolma et al. [14] first identified the compound erastin, which exhibited selective lethality against cancer cells expressing RAS, but it was noted that cells died in a manner different from that typically observed with known programmed cell death. With the continuous development of this research, Dixon et al. [11] first proposed the concept of ferroptosis, based on its distinct morphological characteristics and function in 2012. Ferroptosis was defined as an iron-dependent form of regulated cell death that involves the iron-catalyzed accumulation of lethal lipid peroxides. Under physiological and pathological conditions, cell death is an inevitable and important function in biological processes and marks the end of cell life. Cells undergoing ferroptosis have different morphological and metabolic characteristics from other known forms of cell death (such as apoptosis, necrotizing apoptosis, and pyroptosis) [15]. Morphologically, ferroptosis mainly occurs in cells, presented as decreased mitochondrial volume, decreased or no mitochondrial cristae, and increased bilayer membrane density; however, the nuclear size remains unchanged [16]. Ferroptosis is regulated by many aspects of iron metabolism, including iron absorption, transport, storage, and utilization (section 4.1) [17]. In addition, ferroptosis-inducing factors can affect different pathways of glutathione peroxidase directly or indirectly [18], resulting in a decreased capacity of antioxidants and accumulation of lipid reactive oxygen species (ROS) in cells, culminating in oxidative cell death. Thus, ferrotropis regulation is closely related to the metabolism of iron, lipids, amino acids, and glutathione. Evidence shows that Abnormal ferroptosis is closely related to the occurrence and progression of various diseases, including metabolic diseases, such as T2DM. Over the past decade, an increasing number of studies have supported the view that ferroptosis plays an important pathophysiological role in the occurrence and development of T2DM and its complications [19–22].

## Iron metabolism and glucose homeostasis

**Fig. 1:**
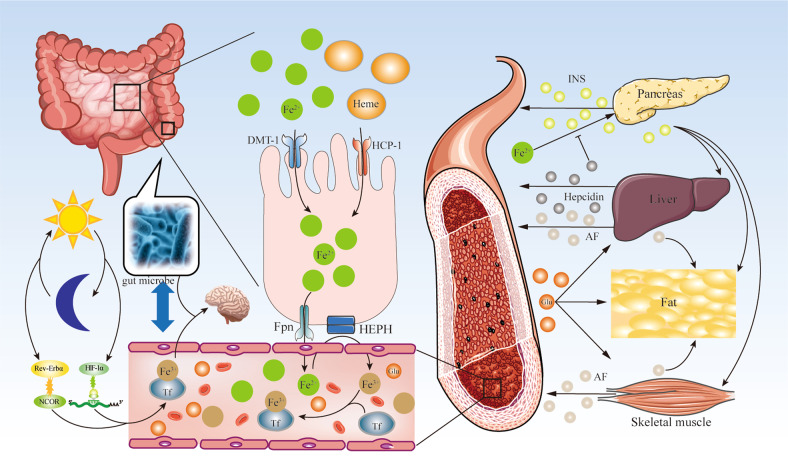
Flow chart of the study. Iron metabolism. Iron is absorbed by intestinal cells in the form of free divalent iron (Fe^2+^) or heme iron (Heme) in the intestine. Fe^2+^ is absorbed by intestinal cells through divergent metal transporter 1 (DMT-1). Heme iron is transported to intestinal cells through heme carrier protein 1 (HCP-1). Fe^2+^ is released into the blood capillary through ferroportin (Fpn), oxidized into free ferric iron (Fe^3+^) by hephaestin (HEPN), combined with transferrin (Tf) in the circulation, and transported to organs and tissues. Fe^3+^ enters the pancreas, liver, fat, and skeletal muscle to regulate blood glucose (Glu) homeostasis. Pancreas β Cells release insulin (INS) in response to the stimulation of Glu. INS affects the liver, fat, and skeletal muscles. The liver and skeletal muscles release adipose factor (AF), which regulates adipose tissue metabolism. On the other hand, iron metabolism also influences the composition of the gut microbiota, which may affect cognitive through the gut-brain axis. Iron affects circadian glucose metabolism via the regulation of the interaction of nuclear receptor subfamily 1 group d member 1 (Rev-Erbα) with its co-suppressor, nuclear receptor corepressor 1 (NCOR). iron also participates in the regulation of β-cell function mediated by HIF-1α in circadian rhythms.

In the human being, iron binds to transferrin in the plasma. Transferrin limits the production of free radicals and is the main carrier of iron to cells; the transferrin receptor (TfR) binds to iron to form a trivalent iron complex (Tf-Fe^3+^) and is transported to a tissue cell that contains a transferrin receptor [8]. Presently, the evaluation of plasma ferritin concentration is a clinically useful method for measuring iron storage. In the human body, it is a sign of iron overload that the plasma transferrin is greater than 45%. And iron overload is a known risk factor for T2DM [23]. The initial description of IR may be in patients with hereditary hemochromatosis (HH). The mechanism of HH is iron deposition in pancreas β cells, and HH induces cell death which leads to diabetes [4]. A systematic review showed that the TfR to ferritin ratio was negatively related to the risk of T2DM and that plasma transferrin may be related to diabetes development directly or indirectly [24]. A cohort study showed that higher plasma serum ferritin levels were significantly associated with an increased risk of T2DM. These results support iron intake and storage as an indicator of T2DM, which could potentially allow for early diagnosis in clinical practice [25]. Due to the relationship between iron metabolism and glucose homeostasis, maintaining normal iron metabolism is a key factor in maintaining blood glucose stability. Below, we discuss the key processes of iron metabolism in maintaining blood glucose homeostasis, in different tissues under physiological and pathological conditions (Fig. 1).

### Dietary iron uptake

The main source of iron is diet [26]. Upon intake, iron is primarily absorbed by intestinal cells in the form of free Fe^2+^ or heme iron [27]. Free Fe^2+^ in the intestine is absorbed by intestinal cells through divergent metal transporter 1 (DMT-1) [28]. Heme iron is transported to the intestinal cells through heme carrier protein 1 (HCP-1) [29]. In the basolateral membrane of the intestinal epithelium, Fe^2+^ is released into the blood capillary through ferroportin [30], oxidized by hephaestin, combined with transferrin in the circulation, and transported to various organs and tissues.

### Insulin secretion

**Fig. 2:**
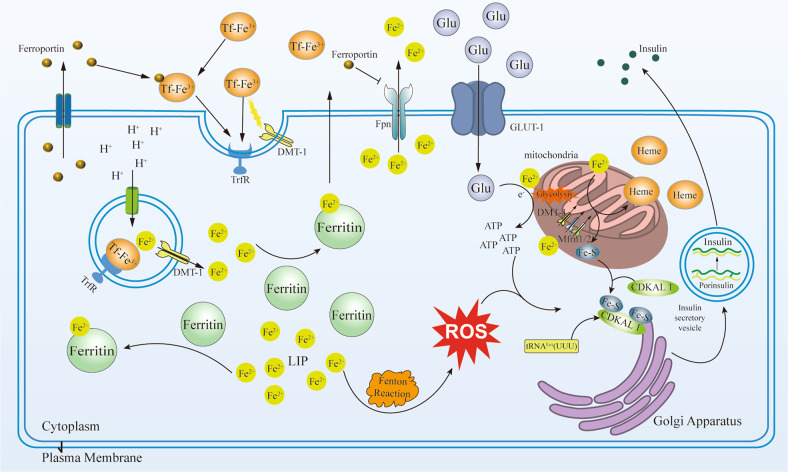
Iron metabolism in pancreatic β cells. Iron metabolism in regulating insulin secretion in pancreatic β cells. Fe^3+^ combined with transferrin (Tf) into Tf-Fe^2+^ in the circulation. Tf-Fe^2+^ binds to the transferrin receptor (TrfR) on the cell surface, and the receptor complex is endocytosed with the divergent metal transporter 1 (DMT-1). Inside the endosome, Fe^3+^ is reduced to Fe^2+^ and released into the labile iron pool (LIP). Ferritin combines with Fe^2+^ in LIP to regulate the concentration of Fe^2+^ in cells. In addition, Fe^2+^ is discharged from cells through ferroportin (Fpn). The pancreas β cells, and hepatocyte can release hepcidin, which can induce Tf internalized and inhibit the activity of Fpn. Glucose (Glu) enters the pancreas β cells via glucose transporter 2 (GLUT-2) and performs glycolysis before entering the mitochondria, which leads to increased ATP production. Fe^2+^ promotes the production of reactive oxygen species (ROS) through the Fenton reaction. Iron exchange in the mitochondria is mediated by DMT-1 and classical mitoferrins (Mfrn) 1 and 2, which can be incorporated into the electron transport chain and produce more ATP under the stimulation of glucose. Fe^2+^ participates in Fe-S cluster biosynthesis in mitochondria. The Fe-S cluster promotes Cdkal1 catalytic metabolism of t^6^A37 in tRNA^Lys^UUU to ms^2^t^6^A37 and enables the normal processing of proinsulin into insulin.

Iron plays an important role in insulin secretion function in pancreas β cells. Tf-Fe^3+^ is absorbed into pancreas β cells through DMT-1 [31]. The pancreas β cells strictly control iron homeostasis, to avoid excessive harmful free iron. Consequently, Fe^2+^ is reserved in the labile iron pool (LIP), where iron is sequestered by ferritin (a unique cytoplasmic iron storage protein) [32]. Fe^2+^ is bound in ferritin for the synthesis of iron-dependent proteins in the cytoplasm or mitochondria [33]. In extracellular, the pancreas β cells release hepcidin, which binds transferrin and induces their internalized [34, 35]. Studies have shown that transferrin mediates a positive feedback mechanism for iron regulation in the process of glucose-stimulated insulin secretion [36]. Fe^2+^ in the Labile iron pool (LIP) is involved in insulin secretion, via three pathways. Although iron can be found in almost all intracellular organelles, iron is predominantly consumed by mitochondria which is the primary source of cellular iron metabolism. Synthesis of heme and Fe-S clusters, used for electron transport proteins, occurs in the mitochondria. Under high-glucose stimulation, glucose enters the pancreas β cells via glucose transporter 2 (GLUT-2) [37] and undergoes glycolysis before entering the mitochondria, which leads to increased ATP production. Iron exchange in the mitochondria is mediated by DMT-1 and classical mitoferrins (Mfrn) 1 and 2 [38], which are incorporated into the electron transport chain and produce ATP under the stimulation of glucose. An increase in the ATP to ADP ratio triggers insulin secretion. In addition, Fe^2+^ promotes ROS production via the Fenton reaction, which is regarded as an amplified signal for insulin secretion [39, 40]. Recent research has shown that Fe^2+^ participates in Fe-S cluster biosynthesis. The Fe-S cluster promotes the Cdkal1 catalytic metabolism of t^6^A37 in tRNA^Lys^UUU to ms^2^t^6^A37 and enables the normal processing of proinsulin into insulin [41]. The Fe-S cluster is an iron mitochondrial chaperone, expressed in pancreas β cells and stimulated by hyperglycaemic disease [42]. Iron is a cofactor of several enzymes and an important component of the Fe-S cluster, participating in insulin secretion as well as in the proliferation and differentiation of β cells. Proinsulin translation in pancreas β cells requires the activity of the Fe-S cluster enzyme, CDKAL1. CDKAL1 dysfunction leads to lysine codon misreading in proinsulin and impairs proinsulin processing, thereby reducing insulin concentration and secretion [41]. A high-glucose environment will lead to an increase in extracellular hepcidin concentration and inhibit the excretion of intracellular Fe^2+^ through ferroportin [43]. It was found that upon glucose stimulation, islet tissue from iron-deficient mice showed impaired insulin release. In glucose depleted environment, human pancreas β cells upregulate the expression of the TfR [44]. However, excess TfR may be toxic due to excessive activation of the oxidation pathway and ROS accumulation. The redox-active iron form (Fe^2+^) oxidizes lipids, in the Fenton reaction, which results in the production of a large amount of ROS, further oxidation of DNA and proteins are mediated by ROS, which results in insulin synthesis and secretion reduction, and ultimately apoptosis [45] (Fig. 2).

### Adipocyte metabolism

Iron is an important regulator of energy metabolism, primarily in adipose tissue. Adipose tissue has specific dynamic characteristics that are helpful in regulating the steady state of carbohydrate and lipid metabolism in the human body. Adipose tissue regulates metabolism by responding to signaling molecules, such as adipokines produced by other metabolic tissues (for example by crosstalk between the liver and skeletal muscle) [46]. Mitochondrial dysfunction in the adipose tissue is a key determinant of the etiology of type 2 diabetes [47]. Iron deficiency and iron overload are considered important causes of chronic metabolic diseases (such as T2DM or obesity) [48]. Studies have shown that the expression of ACO1 is positively related to adipogenic markers in adipose tissue. Although the mechanism of ACO1 and transferrin affecting adipose tissue metabolism is unclear, ACO1 gene expression is significantly related to gene expression of proliferator-activated receptor-gamma coactivator 1-beta (PGC-1β), which is critical to the mitochondrial function of adipose tissue [49]. Therefore, further research is required to clarify the relationship between the mitochondrial oxidation capacity of adipocytes and iron regulation. Iron is the key regulator of mitochondrial biogenesis. As an important component of the Fe-S cluster, iron is necessary for mitochondrial oxidation regulation [50]. It was found that reducing Mfrn1/2 in mitochondria reduced the mitochondrial iron content, oxygen consumption rate, and ATP level in fat cells, which lead to a reduction in lipogenic gene expression and lipid synthesis during lipogenic differentiation [51]. Studies have shown that mice fed with rich iron diets showed upregulation of IR-related adipokines [52]. In addition, the intervention of rat adipocytes with excessive iron leads to reduced glucose transport, after insulin stimulation [53]. Adipocytes are regulated by various cytokines, such as leptin and adiponectin. Studies have shown that leptin and adiponectin levels are reduced in the adipocytes of mice fed a high-iron diet. Clinical experiments have shown that patients with T2DM have higher transferrin and lower adiponectin levels than healthy individuals [54]. Therefore, abnormal iron homeostasis leads to changes in the levels of various fat factors, eventually leading to lipid metabolism disorders and IR.

### Liver metabolism

The relationships among iron metabolism, T2DM, liver function, and liver injury are complex [26]. Hepatocytes play a dual role in iron metabolism: they serve as the main site of iron storage [55] and regulate blood iron content by secreting regulatory hormones, such as hepcidin [56], which controls plasma iron content by binding to transferrin in intestinal epithelial cells and iron circulating macrophages. The binding of transferrin to hepcidin triggers transferrin degradation, thereby reducing transferrin levels. Hepcidin expression is primarily regulated by the BMP-SMAD signaling pathway [57]. In addition, hepatocyte metabolism is regulated by iron. Studies have shown that the iron-sequestering ferritin H chain (FTH) is synthesized in hepatocytes to limit iron-induced hepatic glucose-6-phosphatase (G6Pase) expression and oxidative inhibition. FTH maintains endogenous glucose production, through hepatic gluconeogenesis, which is necessary for hypoglycemic prevention [58].

Studies have shown that iron overload causes IR, which is the risk factor in T2DM and non-alcoholic fatty liver disease (NAFLD) [59, 60]. In multiple models of IR, researchers have found that iron overload leads to the development IR. It was shown that hepatic gluconeogenesis is increased in mouse models of hereditary haemochromatosis [61]. In db/db mice, iron overload aggravates IR and increases hepatic gluconeogenesis [62]. In hypoxia and iron deficiency mouse models, iron restriction caused hypoglycemic, in part due to reduced hepatic gluconeogenesis, possibly due to the activation of the hypoxia-sensing pathway [63]. In the context of the functional interplay between iron metabolism and liver gluconeogenesis, studies have shown that iron can alter the circadian rhythm of hepatic glucose production and affects liver gluconeogenesis (section 2.6) [64]. Inappropriate hepcidin synthesis has been shown to play a role in the pathogenesis of T2DM and its complications. Insufficient hepcidin expression results in iron overload, which triggers ROS synthesis which in turn plays a major role in the pathogenesis of β cell exhaustion and IR-mediated T2DM. Increased hepcidin expression leads to increased intracellular iron sequestration and is associated with T2DM complications [65].

### Gut microbiota

Studies have shown that progressive iron storage and T2DM development, in obese patients, causes aging, and affect the brain microstructure and function. Iron metabolism also influences the composition of the gut microbiota, which is also known to affect cognition via the gut-brain axis [66]. Gut microbiota has emerged as an important risk factor for T2DM and obesity [67]. Therefore, these results suggest a link between iron metabolism, the composition of gut microbiota, and the development of T2DM [66, 68].

### Circadian rhythms

Luconeogenesis is usually suppressed during feeding periods and enhanced during fasting. Circadian rhythm disruption is associated with T2DM, both in experimental animal models and humans, and researchers have found an elevated risk of T2DM in night-shift workers compared to normal individuals [69]. Based on the above findings, researchers have discovered that iron could alter the circadian rhythms of hepatic glucogenesis [64]. Dietary iron regulates circadian glucose metabolism through heme-mediated regulation of the interaction of nuclear receptor subfamily 1 group d member 1 (Rev-Erbα) with its co-suppressor, nuclear receptor corepressor 1 (NCOR). In addition, it was found that iron participates in the regulation of β cell function, mediated by biological clock-based mechanisms driven by HIF-1α. Glucose metabolism and insulin release in β cells are controlled by this mechanism [70, 71], mainly based on the HIF-1α, which can bind promoter regions of clock genes and control their transcription [72]. Some iron-related genes are transcriptionally regulated by clock genes [73, 74], which regulate circadian rhythms and iron homeostasis.

## Ferroptosis in T2DM development

Ferroptosis is a newly discovered process of non-apoptotic cell death that is dependent on excess cellular iron uptake [6]. Ferroptosis is associated with reduced mitochondrial volume, and unlike known programmed death pathways, it is not associated with organelle swelling, chromatin condensation, or autophagy. Instead, it is characterized by iron accumulation, lipid peroxidation, and reduced glutathione peroxidase 4 (Gpx4) expression [8, 26, 75]. In vivo studies have found a potential association between excessive iron storage and T2DM [76]. This partly reveals a correlation between iron and T2DM, which is closely associated with the development of IR [61]. Therefore, the prevailing view is that the higher the iron storage, the higher the risk of developing T2DM. However, this has not yet been effectively demonstrated. Reducing iron storage levels in vivo, has resulted in improved insulin secretion and peripheral tissue insulin sensitivity, which lead to better control of blood glucose and T2DM condition improvement [77]. Herein, we discuss the ferroptosis pathways and molecules involved in the development of T2DM and its complications. The link between ferroptosis and the development of T2DM and its complications has not yet been fully elucidated.

### Ferroptosis and glucose metabolism disorder

**Fig. 3:**
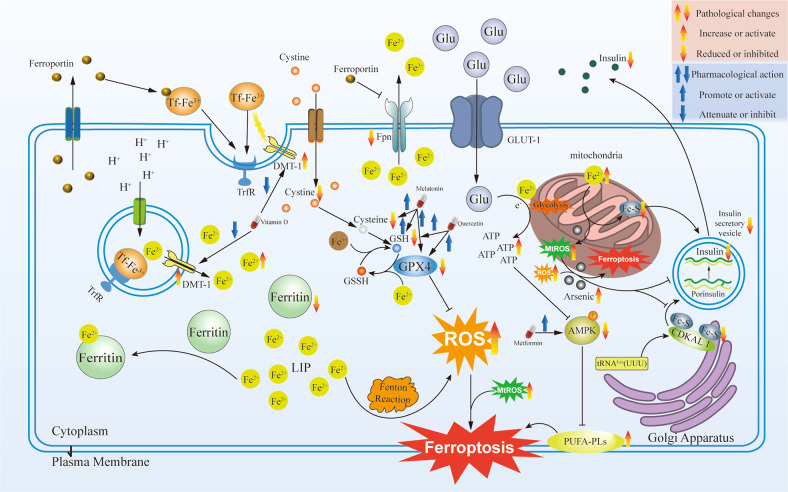
Pharmacological target for treating ferroptosis in pancreatic β cells. Ferroptosis in pancreatic β cells and pharmacological target mechanisms of different drugs. Pancreatic β cells express low levels of antioxidant enzymes, such as superoxide dismutase (SOD), glutathione (GSH) peroxidase, and glutathione peroxidase 4 (Gpx4). The Fe2+ in the labile iron pool (LIP) promotes reactive oxygen species synthesis through the Fenton reaction, leading to the accumulation of reactive oxygen species (ROS). External factors will cause mitochondria damage and produce an excess of mitochondrial ROS (MtROS). ROS and MtROS lead to ROS-dependent autophagy and ferroptosis, and cause intracellular iron increased. Iron in mitochondria accumulation will cause the lack of Fe-S clusters, which could lead to ROS increase in the mitochondria. The lack of Fe-S cluster and the increase of ROS will reduce the synthesis and secretion of insulin. Metformin, Quercetin, Melatonin, and Vitamin D effect on different targets to reduce the possibility of ferroptosis in pancreatic β cells.

It is well known that one of the main antioxidant protective enzymes in cells is GPx4, whose lipid peroxide reduction activity plays a crucial role in protecting cells from iron-induced damage and death. Several studies have shown that pancreatic β cells are predisposed to ferroptosis (Fig. 3). Study was shown that pancreatic β cells express low levels of antioxidant enzymes, such as superoxide dismutase (SOD), glutathione (GSH) peroxidase and catalase [78]. Thus, pancreatic β cells are susceptible to oxidative stress. In pancreatic β cells, the Fe^2+^ in the labile iron pool (LIP) promotes ROS synthesis, via the Fenton reaction. ROS accumulation triggers several ferroptosis. It was shown that the glucose-stimulated insulin secretion (GSIS) capacity of pancreatic β cells was significantly reduced when treated with erastin, in vitro. In contrast, pretreatment with the ferroptosis inhibitors, Fer-1 or DFO, reversed the damage caused by GSIS [79]. External factors (e.g. chronic arsenic exposure) cause mitochondrial damage and produce excess mitochondrial ROS (MtROS), which leads to MtROS-dependent autophagy and ferroptosis, resulting in an increase in intracellular iron. This results in the increased production of Fe2^+^ in pancreatic β cells, and impaired insulin secretion. It was experimentally verified that MtROS-mediated pathway blocking promotes pancreatic β cell insulin secretion [80]. An Abnormal Fe-S cluster content in cells can easily lead to ferroptosis [81]. The Fe-S cluster regulates iron homeostasis in mitochondria. Iron accumulation in the mitochondria causes a lack of Fe-S clusters, which leads to increased ROS levels in the mitochondria [82], followed by ferroptosis due to lipid peroxide accumulation [83].

### Ferroptosis in diabetic macroangiopathy

Theoretically, increased iron availability may contribute to diabetic macrovascular disease improvement because free iron has adverse effects on the endothelium [84] and accelerates the development of atherosclerosis [85]. In animal experiments, mice fed with an iron-deficient diet had a reduced incidence of atherosclerotic lesions [86], and iron overload led to a reduction in atherosclerosis [87]. Inhibition of iron-catalyzed oxidative reactions by Deferoxamine (DFX) restores the dilation of the coronary microcirculation and a normal match between myocardial metabolic demand and coronary blood flow in patients with T2DM [88].

### Ferroptosis in diabetic microangiopathy

Early development and accelerated course of diabetic nephropathy have been observed in patients with thalassemia, which is a recognized condition of iron overload [89]. Increased levels of iron in lysosomal proximal renal tubules have been observed in patients with diabetic nephropathy [90]. This observation is related to mutations in HH that appear to predict DN development of diabetic nephropathy [91]. Recent studies suggest that ferroptosis may enhance diabetes nephropathy and impair the renal tubule in diabetes models, via the HIF-1α/HO-1 pathway [92]. Iron increases diabetic kidney injury by increasing oxidative/nitrifying stress and decreasing antioxidant capacity. In addition, iron may be a potential cofactor in diabetic nephropathy, and strict control of iron is therefore important in the diabetic state [93].

### Ferroptosis in diabetic neuropathy

Experimental studies have shown that DFR administration restores motor and sensory nerve conduction velocity, and improves neural blood flow [94]. Several studies have shown a direct beneficial effect of reducing blood glucose and HbA1c levels, after treatment with high-iron concentrations. The number of pro-inflammatory M1 macrophages was reduced in the neural sections and the number of anti-inflammatory M2 macrophages was increased in db/db mice (fed a high-concentration iron diet). These results confirm and extend the finding in STZ diabetic rats [95], suggesting that dietary non-iron supplements may partially prevent the development of peripheral diabetic neuropathy (PDN) [96].

## Ferroptosis as a promising treatment target

Ferroptosis is one of the reasons for the onset of T2DM and its complications; thus, ferroptosis is a very promising therapeutic target for the treatment and prevention of T2DM and metabolic diseases. In this section, we summarize some molecules that can inhibit ferroptosis and discuss the use of these molecules in the different metabolic pathways of ferroptosis (Table 1).

**Table 1:** The potential drugs or molecules of ferroptosis-targeted.

Intervention	subjects	Potential mechanism	References
Metformin	*AMPKα1/α2*^*L/L*^ mice	AMPK^a^	Lee et al. [100]
Quercetin	*C57BL/6J* mice	GSH, GPX4^a^, Fe^b^	Li et al. [106]
Melatonin	*C57BL/6J* mice	Nrf2/ARE signal pathway, HO-1, NQO1^a^	Long et al. [109, 110, 113]
vitamin D	*ZDF* rats	DMT1, NF-κB^b^	Zhao et al. [114]
L6H21	Prediabetic rats	MD2, toll-like receptor 4^b^	Sumneang et al. [115]
M1	*db/db* mice	Fe-S clusters^b^	Marjault et al. [117]

^a^The level is higher than before the intervention.

^b^The level is lower than before the intervention.

### Metformin

Metformin, a biguanide, has been used as the first-line treatment for T2DM for several decades. It has been reported to regulate cellular energy homeostasis by inducing the AMPK signaling pathways [97]. Its basic pharmacological effects include hepatic gluconeogenesis inhibition, glucose uptake promotion, and insulin sensitivity promotion in peripheral tissues [98]. The LKB1/AMPK signaling pathway plays an important role in glucose homeostasis [99]. A previous study showed that LKB1 and its downstream AMP-activated protein kinase (AMPK) blocked ferroptosis by inhibiting the phosphorylation of both acetyl-CoA carboxylase 1 (ACC1) and FAS [100]. Metformin protects cells by activating the AMPK pathway, regulating metabolism, and protecting them from degradation and pathogenic changes at the molecular level. Therefore, we propose that the ability of metformin to improve T2DM is associated with the inhibition of ferroptosis and the reduction of IR. In addition, supplementation of vascular smooth muscle cells (VSMCs) with metformin can enhance the antioxidant capacity of VSMCs, inhibit ferroptosis, and attenuate hyperlipidemia-related vascular calcification through the activation of Nrf2 signaling [101].

### Quercetin

Quercetin, one of the most widely distributed flavonoids, has been reported to have a large number of attractive pharmacological efficacy in epidemiological investigations, including T2DM risk reduction [102]. Quercetin is a natural inhibitor or regulator of iron metabolism and is beneficial in improving diseases caused by iron overload [103–105]. Studies have shown that quercetin treatment significantly restores GSH content and SOD activity in pancreas β cells. The results indicate that quercetin has a potential beneficial effect on T2DM, and functions by inhibiting pancreas β cells ferroptosis, highlighting the promising curative effect of quercetin in T2DM [106].

### Melatonin

Many studies have shown that melatonin is a potent endogenous antioxidant [107], which also indirectly stimulates certain antioxidant enzymes such as SOD and Gpx4 [108]. Recent studies have shown that melatonin reduces diabetic kidney injury and exerts neuroprotective effects by activating the Nrf2/HO-1 pathway and increasing the levels of antioxidant enzymes HO-1 and NAD(P)H dehydrogenase [quinone] 1 (NQO1) [109, 110]. Epidemiological studies have shown that the incidence of osteoporotic fractures increases in T2DM patients compared with healthy populations [111, 112]. High glucose induces ferroptosis via increased ROS/lipid peroxidation/glutathione depletion in type 2 diabetic osteoporosis. Melatonin significantly reduced ferroptosis and improved the osteogenic capacity of MC3T3-E1 cells by activating the Nrf2/HO-1 pathway in vivo and in vitro [113].

### Vitamin D

Studies have shown that iron overload in the pancreas contributes to T2DM pathogeneses. Vitamin D can inhibit ferroptosis in diabetic pancreatic β cells through NF-κB-DMT1 signaling, which is a potential protective drug in the development of T2DM [114].

### Other compounds affecting ferroptosis in T2DM

Systemic inflammation is mainly caused by activation of the myeloid differentiation factor 2 (MD2)/toll-like receptor 4 complex, a key mediator of left ventricular dysfunction in prediabetes. Study was shown that in obese mice the MD2 inhibitor L6H21 effectively reduced systemic inflammation, and L6H21 provides cardioprotective efficacy in a dose-dependent manner, by reducing apoptosis and ferroptosis [115]. Furthermore, Ze450 has been shown to be protective against cellular peroxidation, where Ze450 retains mitochondrial function and integrity by inhibiting ferroptosis. Thus, promoting the ability of cells to recover from oxidative stress both in vitro and in vivo is a therapeutic opportunity for metabolic diseases such as T2DM [116]. A novel molecule, M1, was found to enhance the lability of the Fe-2S clusters of mNT and NAF-1 proteins, reduced mitochondrial iron and ROS accumulation, and successfully treated diabetic mice [117].

### Limitations of ferroptosis-targeted agents

A growing number of evidence supports the role of ferroptosis in the initiation and progression of various metabolic diseases such as T2DM. However, several questions need to be addressed before the therapeutic potential of ferroptosis-targeted agents can be clinically evaluated [17]. What are the crucial safeguarding mechanisms for ferroptosis in diabetes? Which are the reliable biomarkers for predicting ferroptosis in metabolic disease? Plasma ferritin concentration is currently used as a ferroptosis biomarker in preclinical studies. However, it is non-specific and is present in other types of cell death and several pathological conditions. The lack of ferroptosis-specific biomarkers has been a long-standing bottleneck, limiting the development of ferroptosis-targeted clinical applications. Finally, how does the interplay between ferroptosis and other forms of cell death affect the development of metabolic diseases? To date, no clinical trials have investigated ferroptosis-specific inhibitors for metabolic disease treatment. Most of the research uses selective inhibition of ferroptosis, which has been shown to substantially improve pancreatic β cells function in various animal models. Large population-based datasets are urgently needed to determine whether selectively blocking ferroptosis can improve T2DM and its complications.

## Conclusion

The relationship between iron metabolism and glucose homeostasis is now widely recognized. Iron has been shown to affect glucose homeostasis in organs and cells, such as pancreas β cells, hepatocytes, and adipose tissue. In addition, iron metabolism is related to the brain-gut axis and circadian rhythm. Iron metabolism disorders result in insufficient insulin secretion and IR; however, the relationship between iron metabolism and T2DM and its complications was unclear until the discovery of ferroptosis. Excess levels of free reactive iron cause tissue damage and oxidative cell death. Different drugs and compounds for lipid peroxidation have been widely studied for the treatment of T2DM induced by iron overload. However, there is a lack of clinical research at this stage and preclinical research is paving the way for the development of effective ferroptosis-specific antagonists for the clinical treatment of T2DM.

## Data Availability

All data generated or analyzed during this study are included in this published article.

## References

[1] Forbes A (2009). Iron and parenteral nutrition. Gastroenterology.

[2] Prá D, Franke SI, Henriques JA, Fenech M (2012). Iron and genome stability: an update. Mutat Res.

[3] Hentze MW, Muckenthaler MU, Galy B, Camaschella C (2010). Two to tango: regulation of mammalian iron metabolism. Cell.

[4] Soe-Lin S, Apte SS, Andriopoulos B, Andrews MC, Schranzhofer M, Kahawita T (2009). Nramp1 promotes efficient macrophage recycling of iron following erythrophagocytosis in vivo. Proc Natl Acad Sci USA.

[5] Jordan JB, Poppe L, Haniu M, Arvedson T, Syed R, Li V (2009). Hepcidin revisited, disulfide connectivity, dynamics, and structure. J Biol Chem.

[6] Dixon SJ, Lemberg KM, Lamprecht MR, Skouta R, Zaitsev EM, Gleason CE (2012). Ferroptosis: an iron-dependent form of nonapoptotic cell death. Cell.

[7] Jiang X, Stockwell BR, Conrad M (2021). Ferroptosis: mechanisms, biology and role in disease. Nat Rev Mol Cell Biol.

[8] Stockwell BR, Friedmann Angeli JP, Bayir H, Bush AI, Conrad M, Dixon SJ (2017). Ferroptosis: a regulated cell death nexus linking metabolism, redox biology, and disease. Cell.

[9] International Diabetes Federation, “IDF Diabetes Atlas, 9th edn.” Brussels, Belgium, http://www.diabetesatlas.org.

[10] Backe MB, Moen IW, Ellervik C, Hansen JB, Mandrup-Poulsen T (2016). Iron Regulation Of Pancreatic Beta-cell Functions And Oxidative Stress. Annu Rev Nutr.

[11] Gao H, Jin Z, Bandyopadhyay G, Wang G, Zhang D, Rocha K (2022). Aberrant iron distribution via hepatocyte-stellate cell axis drives liver lipogenesis and fibrosis. Cell Metab.

[12] Zhang Z, Funcke JB, Zi Z, Zhao S, Straub LG, Zhu Y (2021). Adipocyte iron levels impinge on a fat-gut crosstalk to regulate intestinal lipid absorption and mediate protection from obesity. Cell Metab.

[13] Fernández-Real JM, McClain D, Manco M (2015). Mechanisms linking glucose homeostasis and iron metabolism toward the onset and progression of type 2 diabetes. Diabetes care.

[14] Dolma S, Lessnick SL, Hahn WC, Stockwell BR (2003). Identification of genotype-selective antitumor agents using synthetic lethal chemical screening in engineered human tumor cells. Cancer Cell.

[15] Li J, Cao F, Yin HL, Huang ZJ, Lin ZT, Mao N (2020). Ferroptosis: past, present and future. Cell Death Dis.

[16] Yang WS, Stockwell BR (2008). Synthetic lethal screening identifies compounds activating iron-dependent, nonapoptotic cell death in oncogenic-RAS-harboring cancer cells. Chem Biol.

[17] Fang X, Ardehali H, Min J, Wang F (2023). The molecular and metabolic landscape of iron and ferroptosis in cardiovascular disease. Nat Rev Cardiol.

[18] Friedmann Angeli JP, Schneider M, Proneth B, Tyurina YY, Tyurin VA, Hammond VJ (2014). Inactivation of the ferroptosis regulator Gpx4 triggers acute renal failure in mice. Nat Cell Biol.

[19] Chen L, Yin Z, Qin X, Zhu X, Chen X, Ding G (2022). CD74 ablation rescues type 2 diabetes mellitus-induced cardiac remodeling and contractile dysfunction through pyroptosis-evoked regulation of ferroptosis. Pharm Res.

[20] Tang W, Li Y, He S, Jiang T, Wang N, Du M (2022). Caveolin-1 alleviates diabetes-associated cognitive dysfunction through modulating neuronal ferroptosis-mediated mitochondrial homeostasis. Antioxid Redox Signal.

[21] Ye H, Wang R, Wei J, Wang Y, Zhang X, Wang L (2022). Bioinformatics analysis identifies potential ferroptosis key gene in type 2 diabetic islet dysfunction. Front Endocrinol (Lausanne).

[22] Krümmel B, von Hanstein AS, Plötz T, Lenzen S, Mehmeti I (2022). Differential effects of saturated and unsaturated free fatty acids on ferroptosis in rat β-cells. J Nutr Biochem.

[23] Simcox JA, McClain DA (2013). Iron and diabetes risk. Cell Metab.

[24] Liu J, Li Q, Yang Y, Ma L (2020). Iron metabolism and type 2 diabetes mellitus: a meta-analysis and systematic review. J Diabetes Investig.

[25] Díaz-López A, Iglesias-Vázquez L, Pallejà-Millán M, Rey Reñones C, Flores Mateo G, Arija V (2020). Association between iron status and incident type 2 diabetes: a population-based cohort study. Nutrients.

[26] Hansen JB, Moen IW, Mandrup-Poulsen T (2014). Iron: the hard player in diabetes pathophysiology. Acta Physiol (Oxf).

[27] Andrews NC, Schmidt PJ (2007). Iron homeostasis. Annu Rev Physiol.

[28] Gunshin H, Fujiwara Y, Custodio AO, Direnzo C, Robine S, Andrews NC (2005). Slc11a2 is required for intestinal iron absorption and erythropoiesis but dispensable in placenta and liver. J Clin Invest.

[29] Evstatiev R, Gasche C (2012). Iron sensing and signalling. Gut.

[30] Donovan A, Lima CA, Pinkus JL, Pinkus GS, Zon LI, Robine S (2005). The iron exporter ferroportin/Slc40a1 is essential for iron homeostasis. Cell Metab.

[31] Koch RO, Zoller H, Theuri I, Obrist P, Egg G, Strohmayer W (2003). Distribution of DMT 1 within the human glandular system. Histol Histopathol.

[32] Marku A, Galli A, Marciani P, Dule N, Perego C, Castagna M (2021). Iron metabolism in pancreatic beta-cell function and dysfunction. Cells.

[33] Gkouvatsos K, Papanikolaou G, Pantopoulos K (2012). Regulation of iron transport and the role of transferrin. Biochim Biophys Acta.

[34] Nemeth E, Tuttle MS, Powelson J, Vaughn MB, Donovan A, Ward DM (2004). Hepcidin regulates cellular iron efflux by binding to ferroportin and inducing its internalization. Science.

[35] Kulaksiz H, Fein E, Redecker P, Stremmel W, Adler G, Cetin Y (2008). Pancreatic beta-cells express hepcidin, an iron-uptake regulatory peptide. J Endocrinol.

[36] Aigner E, Felder TK, Oberkofler H, Hahne P, Auer S, Soyal S (2013). Glucose acts as a regulator of serum iron by increasing serum hepcidin concentrations. J Nutr Biochem.

[37] Morgan D, Rebelato E, Abdulkader F, Graciano MF, Oliveira-Emilio HR, Hirata AE (2009). Association of NAD(P)H oxidase with glucose-induced insulin secretion by pancreatic beta-cells. Endocrinology.

[38] Paradkar PN, Zumbrennen KB, Paw BH, Ward DM, Kaplan J (2009). Regulation of mitochondrial iron import through differential turnover of mitoferrin 1 and mitoferrin 2. Mol Cell Biol.

[39] Robson-Doucette CA, Sultan S, Allister EM, Wikstrom JD, Koshkin V, Bhattacharjee A (2011). Beta-cell uncoupling protein 2 regulates reactive oxygen species production, which influences both insulin and glucagon secretion. Diabetes.

[40] Leloup C, Tourrel-Cuzin C, Magnan C, Karaca M, Castel J, Carneiro L (2009). Mitochondrial reactive oxygen species are obligatory signals for glucose-induced insulin secretion. Diabetes.

[41] Santos M, Anderson CP, Neschen S, Zumbrennen-Bullough KB, Romney SJ, Kahle-Stephan M (2020). Irp2 regulates insulin production through iron-mediated Cdkal1-catalyzed tRNA modification. Nat Commun.

[42] Del Guerra S, D’Aleo V, Gualtierotti G, Pandolfi R, Boggi U, Vistoli F (2012). Evidence for a role of frataxin in pancreatic islets isolated from multi-organ donors with and without type 2 diabetes mellitus. Horm Metab Res.

[43] MacDonald MJ, Cook JD, Epstein ML, Flowers CH (1994). Large amount of (apo)ferritin in the pancreatic insulin cell and its stimulation by glucose. FASEB J.

[44] Berthault C, Staels W, Scharfmann R (2020). Purification of pancreatic endocrine subsets reveals increased iron metabolism in beta cells. Mol Metab.

[45] Cooksey RC, Jouihan HA, Ajioka RS, Hazel MW, Jones DL, Kushner JP (2004). Oxidative stress, beta-cell apoptosis, and decreased insulin secretory capacity in mouse models of hemochromatosis. Endocrinology.

[46] Liu Y, Sweeney G (2014). Adiponectin action in skeletal muscle. Best Pract Res Clin Endocrinol Metab.

[47] Kusminski CM, Ghaben AL, Morley TS, Samms RJ, Adams AC, An Y (2020). A novel model of diabetic complications: adipocyte mitochondrial dysfunction triggers massive β-cell hyperplasia. Diabetes.

[48] Rodríguez-Pérez C, Vrhovnik P, González-Alzaga B, Fernández MF, Martin-Olmedo P, Olea N (2018). Socio-demographic, lifestyle, and dietary determinants of essential and possibly-essential trace element levels in adipose tissue from an adult cohort. Environ Pollut.

[49] Enguix N, Pardo R, González A, López VM, Simó R, Kralli A (2013). Mice lacking PGC-1β in adipose tissues reveal a dissociation between mitochondrial dysfunction and insulin resistance. Mol Metab.

[50] Medina-Gómez G (2012). Mitochondria and endocrine function of adipose tissue. Best Pract Res Clin Endocrinol Metab.

[51] Xu W, Barrientos T, Andrews NC (2013). Iron and copper in mitochondrial diseases. Cell Metab.

[52] Dongiovanni P, Ruscica M, Rametta R, Recalcati S, Steffani L, Gatti S (2013). Dietary iron overload induces visceral adipose tissue insulin resistance. Am J Pathol.

[53] Rumberger JM, Peters T, Burrington C, Green A (2004). Transferrin and iron contribute to the lipolytic effect of serum in isolated adipocytes. Diabetes.

[54] Gabrielsen JS, Gao Y, Simcox JA, Huang J, Thorup D, Jones D (2012). Adipocyte iron regulates adiponectin and insulin sensitivity. J Clin Invest.

[55] Pietrangelo A (2016). Iron and the liver. Liver Int.

[56] Camaschella C, Nai A, Silvestri L (2020). Iron metabolism and iron disorders revisited in the hepcidin era. Haematologica.

[57] Nai A, Rubio A, Campanella A, Gourbeyre O, Artuso I, Bordini J (2016). Limiting hepatic Bmp-Smad signaling by matriptase-2 is required for erythropoietin-mediated hepcidin suppression in mice. Blood.

[58] Weis S, Carlos AR, Moita MR, Singh S, Blankenhaus B, Cardoso S (2017). Metabolic adaptation establishes disease tolerance to sepsis. Cell.

[59] Mehta KJ, Farnaud SJ, Sharp PA (2019). Iron and liver fibrosis: mechanistic and clinical aspects. World J Gastroenterol.

[60] Deugnier Y, Bardou-Jacquet É, Lainé F (2017). Dysmetabolic iron overload syndrome (DIOS). Presse Med.

[61] Huang J, Jones D, Luo B, Sanderson M, Soto J, Abel ED (2011). Iron overload and diabetes risk: a shift from glucose to fatty acid oxidation and increased hepatic glucose production in a mouse model of hereditary hemochromatosis. Diabetes.

[62] Altamura S, Müdder K, Schlotterer A, Fleming T, Heidenreich E, Qiu R (2021). Iron aggravates hepatic insulin resistance in the absence of inflammation in a novel db/db mouse model with iron overload. Mol Metab.

[63] Nam H, Jones D, Cooksey RC, Gao Y, Sink S, Cox J (2016). Synergistic inhibitory effects of hypoxia and iron deficiency on hepatic glucose response in mouse liver. Diabetes.

[64] Simcox JA, Mitchell TC, Gao Y, Just SF, Cooksey R, Cox J (2015). Dietary iron controls circadian hepatic glucose metabolism through heme synthesis. Diabetes.

[65] Ambachew S, Biadgo B (2017). Hepcidin in iron homeostasis: diagnostic and therapeutic implications in type 2 diabetes mellitus patients. Acta Haematol.

[66] Fernández Real JM, Moreno-Navarrete JM, Manco M (2019). Iron influences on the Gut-Brain axis and development of type 2 diabetes. Crit Rev Food Sci Nutr.

[67] Sonnenburg JL, Bäckhed F (2016). Diet-microbiota interactions as moderators of human metabolism. Nature.

[68] Mayneris-Perxachs J, Cardellini M, Hoyles L, Latorre J, Davato F, Moreno-Navarrete JM (2021). Iron status influences non-alcoholic fatty liver disease in obesity through the gut microbiome. Microbiome.

[69] Li W, Chen Z, Ruan W, Yi G, Wang D, Lu Z (2019). A meta-analysis of cohort studies including dose-response relationship between shift work and the risk of diabetes mellitus. Eur J Epidemiol.

[70] Boden G, Ruiz J, Urbain JL, Chen X (1996). Evidence for a circadian rhythm of insulin secretion. Am J Physiol.

[71] Perelis M, Marcheva B, Ramsey KM, Schipma MJ, Hutchison AL, Taguchi A (2015). Pancreatic β cell enhancers regulate rhythmic transcription of genes controlling insulin secretion. Science.

[72] Peek CB, Levine DC, Cedernaes J, Taguchi A, Kobayashi Y, Tsai SJ (2017). Circadian clock interaction with HIF1α mediates oxygenic metabolism and anaerobic glycolysis in skeletal muscle. Cell Metab.

[73] Okazaki F, Matsunaga N, Okazaki H, Azuma H, Hamamura K, Tsuruta A (2016). Circadian clock in a mouse colon tumor regulates intracellular iron levels to promote tumor progression. J Biol Chem.

[74] Yang M, Chen P, Liu J, Zhu S, Kroemer G, Klionsky DJ (2019). Clockophagy is a novel selective autophagy process favoring ferroptosis. Sci Adv.

[75] Latunde-Dada GO (2017). Ferroptosis: role of lipid peroxidation, iron and ferritinophagy. Biochim Biophys Acta Gen Subj.

[76] Sun L, Zong G, Pan A, Ye X, Li H, Yu Z (2013). Elevated plasma ferritin is associated with increased incidence of type 2 diabetes in middle-aged and elderly Chinese adults. J Nutr.

[77] Fernández-Real JM, Peñarroja G, Castro A, García-Bragado F, Hernández-Aguado I, Ricart W (2002). Blood letting in high-ferritin type 2 diabetes: effects on insulin sensitivity and beta-cell function. Diabetes.

[78] Wang J, Wang H (2017). Oxidative stress in pancreatic beta cell regeneration. Oxid Med Cell Longev.

[79] Bruni A, Pepper AR, Pawlick RL, Gala-Lopez B, Gamble AF, Kin T (2018). Ferroptosis-inducing agents compromise in vitro human islet viability and function. Cell Death Dis.

[80] Wei S, Qiu T, Yao X, Wang N, Jiang L, Jia X (2020). Arsenic induces pancreatic dysfunction and ferroptosis via mitochondrial ROS-autophagy-lysosomal pathway. J Hazard Mater.

[81] Pain D, Dancis A (2016). Roles of Fe-S proteins: from cofactor synthesis to iron homeostasis to protein synthesis. Curr Opin Genet Dev.

[82] Mena NP, Urrutia PJ, Lourido F, Carrasco CM, Núñez MT (2015). Mitochondrial iron homeostasis and its dysfunctions in neurodegenerative disorders. Mitochondrion.

[83] Du J, Wang T, Li Y, Zhou Y, Wang X, Yu X (2019). DHA inhibits proliferation and induces ferroptosis of leukemia cells through autophagy dependent degradation of ferritin. Free Radic Biol Med.

[84] Lekakis J, Papamicheal C, Stamatelopoulos K, Cimponeriu A, Voutsas A, Vemmos K (1999). Hemochromatosis associated with endothelial dysfunction: evidence for the role of iron stores in early atherogenesis. Vasc Med.

[85] Araujo JA, Romano EL, Brito BE, Parthé V, Romano M, Bracho M (1995). Iron overload augments the development of atherosclerotic lesions in rabbits. Arterioscler Thromb Vasc Biol.

[86] Lee TS, Shiao MS, Pan CC, Chau LY (1999). Iron-deficient diet reduces atherosclerotic lesions in apoE-deficient mice. Circulation.

[87] Kirk EA, Heinecke JW, LeBoeuf RC (2001). Iron overload diminishes atherosclerosis in apoE-deficient mice. J Clin Invest.

[88] Nitenberg A, Ledoux S, Valensi P, Sachs R, Antony I (2002). Coronary microvascular adaptation to myocardial metabolic demand can be restored by inhibition of iron-catalyzed formation of oxygen free radicals in type 2 diabetic patients. Diabetes.

[89] Loebstein R, Lehotay DC, Luo X, Bartfay W, Tyler B, Sher GD (1998). Diabetic nephropathy in hypertransfused patients with beta-thalassemia. The role of oxidative stress. Diabetes Care.

[90] Nankivell BJ, Tay YC, Boadle RA, Harris DC (1994). Lysosomal iron accumulation in diabetic nephropathy. Ren Fail.

[91] Moczulski DK, Grzeszczak W, Gawlik B (2001). Role of hemochromatosis C282Y and H63D mutations in HFE gene in development of type 2 diabetes and diabetic nephropathy. Diabetes Care.

[92] Feng X, Wang S, Sun Z, Dong H, Yu H, Huang M (2021). Ferroptosis enhanced diabetic renal tubular injury via HIF-1α/HO-1 pathway in db/db mice. Front Endocrinol (Lausanne).

[93] Gao W, Li X, Gao Z, Li H (2014). Iron increases diabetes-induced kidney injury and oxidative stress in rats. Biol Trace Elem Res.

[94] Cameron NE, Cotter MA (2001). Effects of an extracellular metal chelator on neurovascular function in diabetic rats. Diabetologia.

[95] Baum P, Kosacka J, Estrela-Lopis I, Woidt K, Serke H, Paeschke S (2016). The role of nerve inflammation and exogenous iron load in experimental peripheral diabetic neuropathy (PDN). Metabolism.

[96] Paeschke S, Baum P, Toyka KV, Blüher M, Koj S, Klöting N (2019). The role of iron and nerve inflammation in diabetes mellitus type 2-induced peripheral neuropathy. Neuroscience.

[97] Zhang CS, Jiang B, Li M, Zhu M, Peng Y, Zhang YL (2014). The lysosomal v-ATPase-Ragulator complex is a common activator for AMPK and mTORC1, acting as a switch between catabolism and anabolism. Cell Metab.

[98] Rena G, Hardie DG, Pearson ER (2017). The mechanisms of action of metformin. Diabetologia.

[99] Hsu SK, Cheng KC, Mgbeahuruike MO, Lin YH, Wu CY, Wang HD (2021). New insight into the effects of metformin on diabetic retinopathy, aging and cancer: nonapoptotic cell death, immunosuppression, and effects beyond the AMPK pathway. Int J Mol Sci.

[100] Lee H, Zandkarimi F, Zhang Y, Meena JK, Kim J, Zhuang L (2020). Energy-stress-mediated AMPK activation inhibits ferroptosis. Nat Cell Biol.

[101] Ma WQ, Sun XJ, Zhu Y, Liu NF (2021). Metformin attenuates hyperlipidaemia-associated vascular calcification through anti-ferroptotic effects. Free Radic Biol Med.

[102] Yao Z, Gu Y, Zhang Q, Liu L, Meng G, Wu H (2019). Estimated daily quercetin intake and association with the prevalence of type 2 diabetes mellitus in Chinese adults. Eur J Nutr.

[103] Bardy G, Virsolvy A, Quignard JF, Ravier MA, Bertrand G, Dalle S (2013). Quercetin induces insulin secretion by direct activation of L-type calcium channels in pancreatic beta cells. Br J Pharm.

[104] Eitah HE, Maklad YA, Abdelkader NF, Gamal El Din AA, Badawi MA, Kenawy SA (2019). Modulating impacts of quercetin/sitagliptin combination on streptozotocin-induced diabetes mellitus in rats. Toxicol Appl Pharm.

[105] Wang Z, Zhai D, Zhang D, Bai L, Yao R, Yu J (2017). Quercetin decreases insulin resistance in a polycystic ovary syndrome rat model by improving inflammatory microenvironment. Reprod Sci.

[106] Li D, Jiang C, Mei G, Zhao Y, Chen L, Liu J (2020). Quercetin alleviates ferroptosis of pancreatic β cells in type 2 diabetes. Nutrients.

[107] Galano A, Reiter RJ (2018). Melatonin and its metabolites vs oxidative stress: from individual actions to collective protection. J Pineal Res.

[108] Zhao Z, Lu C, Li T, Wang W, Ye W, Zeng R (2018). The protective effect of melatonin on brain ischemia and reperfusion in rats and humans: in vivo assessment and a randomized controlled trial. J Pineal Res.

[109] Long T, Yang Y, Peng L, Li Z (2018). Neuroprotective effects of melatonin on experimental allergic encephalomyelitis mice via anti-oxidative stress activity. J Mol Neurosci.

[110] Shi S, Lei S, Tang C, Wang K, Xia Z (2019). Melatonin attenuates acute kidney ischemia/reperfusion injury in diabetic rats by activation of the SIRT1/Nrf2/HO-1 signaling pathway. Biosci Rep.

[111] Rathmann W, Kostev K (2015). Fracture risk in patients with newly diagnosed type 2 diabetes: a retrospective database analysis in primary care. J Diabetes Complicat.

[112] Vestergaard P (2007). Discrepancies in bone mineral density and fracture risk in patients with type 1 and type 2 diabetes-a meta-analysis. Osteoporos Int.

[113] Ma H, Wang X, Zhang W, Li H, Zhao W, Sun J (2020). Melatonin suppresses ferroptosis induced by high glucose via activation of the Nrf2/HO-1 signaling pathway in type 2 diabetic osteoporosis. Oxid Med Cell Longev.

[114] Zhao Y, Mei G, Zhou F, Kong B, Chen L, Chen H (2022). Vitamin D decreases pancreatic iron overload in type 2 diabetes through the NF-κB-DMT1 pathway. J Nutr Biochem.

[115] Sumneang N, Oo TT, Singhanat K, Maneechote C, Arunsak B, Nawara W (2022). nhibition of myeloid differentiation factor 2 attenuates cardiometabolic impairments via reducing cardiac mitochondrial dysfunction, inflammation, apoptosis and ferroptosis in prediabetic rats. Biochim Biophys Acta Mol Basis Dis.

[116] Rabenau M, Dillberger B, Günther M, Krippner S, Butterweck V, Boonen G (2021). Cimicifuga racemosa extract Ze 450 re-balances energy metabolism and promotes longevity. Antioxid (Basel).

[117] Marjault HB, Karmi O, Zuo K, Michaeli D, Eisenberg-Domovich Y, Rossetti G, et al. An anti-diabetic drug targets NEET (CISD) proteins through destabilization of their [2Fe-2S] clusters. Commun Biol. 2022;5:437. 10.1038/s42003-022-03393-x PMC9090738 35538231

